# Healthcare Preparedness: Saving Lives

**DOI:** 10.1089/hs.2016.0090

**Published:** 2017-02-01

**Authors:** Eric Toner

Preparing our hospitals and other healthcare facilities for disasters is a national security priority. Disasters occur nearly every day in the United States, and the frequency is increasing. This includes such diverse events as storms, droughts, wildfires, floods, earthquakes, chemical and industrial accidents, burns, mass shootings and bombings, and epidemics. All sickened or injured people require a well-prepared public health and healthcare system. The number of people killed by disasters depends not only on the severity of the event itself but also on our ability to respond effectively and treat the ill or injured. This is true of all disasters, whether they are natural disasters like earthquakes and hurricanes or manmade disasters like terrorism. It is also true of infectious disease epidemics, whether of natural or manmade origin.

## Healthcare Preparedness Reduces Risk

In theoretical terms, Risk = Threat × Vulnerability × Consequence. Thus, even if we are not successful in reducing the threat of disasters or our vulnerability to them, we can reduce our nation's risk by mitigating the consequences—by preparing our health facilities and communities to treat the sick and protect the well. In the end, healthcare preparedness is about saving lives and reducing the long-term health consequences of disasters.

## Why Government Programs Are Needed

Preparedness activities are expensive and do not produce offsetting revenue. Although they occur daily at the national level, disasters are low-probability events at the local level. The chance that any individual hospital will face a disaster in the next 5 years is small. So in the view of a hospital executive facing tight budgets, it is hard to make the business case for preparedness. We speak of this as the *parking lot conundrum*: Is it better to spend a million dollars on preparedness that might never be needed, or spend it on improving parking that will lead directly to more revenue in the short term? A strictly financial analysis might well favor the parking lot. But although the probability is low for an individual facility, the consequences can be extraordinary, as so vividly demonstrated after Hurricane Katrina^1^ and to a lesser extent in the aftermath of Hurricane Sandy.^2,3^ Getting back to the risk equation above, this means that the risk is higher than it may seem if one is just focusing on the probability. In many ways, preparedness is best thought of as insurance: paying a small regular amount to protect from the severe consequences of a disaster. Even knowing this, many hospitals choose to do only the minimum that is required. Consequently, over the past 15 years most of the progress in healthcare preparedness has come as a result of federal programs and the stricter accreditation requirements of the Joint Commission (the private entity that accredits most hospitals).

## A Brief History of Federal Programs

The National Disaster Medical System (NDMS) has been in existence since the Cold War. It was originally designed to manage large numbers of military casualties from a foreign battlefield. It consists of 3 components: deployable teams of emergency medical responders, a network of private hospitals that have agreed to take evacuated disaster victims, and a transportation coordination system. While valuable, the magnitude of resources available from NDMS are insufficient for large-scale events and are dwarfed by the scale of the US healthcare system.^4^

The modern era of healthcare preparedness began in 1996 in response to increasingly frequent terrorism events in the 1990s, such as the 1993 bombing of the World Trade Center in New York City, the 1995 bombing of the Oklahoma City Alfred P. Murrah Federal Building, and the 1995 biological and chemical terror attacks by Aum Shinrikyo in Japan. The Metropolitan Medical Response Program (MMRS) was created at the Department of Health and Human Services (HHS) in 1996 but eventually transferred to the Department of Homeland Security (DHS). The program was designed to prepare communities for an attack by weapons of mass destruction. It created 127 local jurisdictions in which agencies involved in emergency management, law enforcement, EMS, fire, and hazardous material planned together along with healthcare facilities.^5^ The program has been defunded since 2011, although some local MMRS committees continue to exist and provide a basis for regional preparedness efforts.

At the hospital level, significant change occurred in early 2001 with the release of new, much stricter Joint Commission standards for emergency preparedness. These new standards were just being implemented when the 9/11 attacks occurred, followed immediately by the anthrax letters. Within months, HHS Secretary Tommy Thompson established an Office of Public Health Preparedness. Very soon this new office created the National Bioterrorism Hospital Preparedness Program (NBHPP) with a mandate to prepare the country's 5,000 hospitals for what was expected to be an imminent bioattack. Initially, the NBHPP was funded at approximately $500 million per year, and the funds were distributed to hospitals through cooperative agreements with state health departments.

In 2006, the Pandemic and All-Hazards Preparedness Act (PAHPA) was signed, replacing the Office of Public Health Preparedness with the new Office of the Assistant Secretary for Preparedness and Response (ASPR). Following the creation of ASPR, the NBHPP was changed to the Hospital Preparedness Program (HPP), with a broadened mission to address “all-hazard” healthcare preparedness. Over the past 10 years, the HPP has continued to evolve. Increasingly, the focus of the program has been on the development of healthcare coalitions (HCC) designed to foster emergency preparedness collaboration among hospitals and between hospitals and public health agencies, emergency management agencies, and emergency medical services. At the same time, its budget has been cut by approximately 50% from its 2003 levels.

In parallel with the HPP, the Centers for Disease Control and Prevention (CDC) launched the Public Health Emergency Preparedness (PHEP) cooperative agreement in 2002 to better prepare state, local, tribal, and territorial public health departments for public health emergencies. The funding for this program has decreased by a third over the years.^6^

Other federal preparedness programs touch the health sector as well but do not directly affect hospitals. These include, among others:

• DHS's Urban Area Security Initiative (UASI), which funds emergency management activities to build and sustain the capabilities necessary to prevent, protect against, mitigate, respond to, and recover from acts of terrorism in designated high-threat, high-density urban areas.^7^
• The CDC National Strategic Stockpile (SNS), which maintains the nation's emergency supply of drugs, vaccines, and medical supplies for use in a public health emergency.^8^
• CDC's Cities Readiness Initiative (CRI), which funds public health activities in 72 large cities to enable them to quickly receive and distribute emergency items from the SNS following a large-scale public health emergency.^9^
• ASPR's Medical Reserve Corps (MRC) program, which is a national network of local volunteer groups organized to help with aspects of health related to disasters.^10,11^

The newest entry in the spectrum of federal health preparedness initiatives is the Centers for Medicare and Medicaid Services (CMS) preparedness rule finalized on September 16, 2016. This rule establishes emergency preparedness requirements for facilities and suppliers participating in Medicare and Medicaid to prepare for disasters and coordinate with federal, state, and local emergency preparedness systems. This rule comes with no additional funding and little in the way of an enforcement mechanism.^12^

## What We Have Learned

The investment in healthcare preparedness has produced positive results. This has been demonstrated in many disasters in recent years. Hospitals and public health are better prepared than they were before, and collaboration among hospitals, public health departments, emergency management agencies, and EMS has improved. But many challenges remain. Many coalitions are not well developed, and the reductions in federal funding threaten the progress that has been made. Health departments and hospitals struggle to find the money needed to maintain their healthcare coalitions and the essential functions they support, such as regional exercises and joint training, much less expanding their coalitions. Yet, maintaining healthcare coalitions and expanding them is important to the nation's health security.

Coalition building, however, is hard work. Hospitals are fiercely competitive and reluctant to share data and resources. Getting them to work well together has been a challenge. This has been complicated by the emergence of integrated health networks that merge multiple hospitals, physician offices, and other services into one large enterprise. A foundational business principle of these networks is keeping the patients and resources within the networks. This makes local cross-institutional coalition building more challenging. But these are not reasons to shy away from the challenge of coalition building; they are reasons to invest even more in promoting them. By eliminating redundancies and taking advantage of efficiencies of scale, healthcare coalitions are the best way to leverage limited preparedness funds.

This does not mean that healthcare preparedness should be solely a federal responsibility. States, counties, cities, and hospitals themselves have an important stake in having a well-prepared health system. But the investments by hospitals and localities are often less tangible than federal budgets and therefore harder to identify and track. Research is need to quantify the nonfederal contributions to healthcare preparedness.

## Recommendations

➢ **HPP funding should be substantially increased to account for the many new entities that will be encouraged to join healthcare coalitions.**

A much broader segment of health-related entities are likely to be motivated to join healthcare coalitions because of the new CMS preparedness rule. This new unfunded mandate, while good for preparedness, creates additional financial burdens on these organizations and on the coalitions. These new costs, in addition to the already inadequate funding for maintaining coalitions, suggests that HPP funding should be increased by 50% to $375 million, which is still $125 million less than the program's funding at its peak.

The experiences of Hurricanes Katrina and Sandy demonstrate that many more components of the health sector than just hospitals and public health departments play important roles in the resilience of the healthcare system. This includes medical clinics, home health services, dialysis centers, behavioral health providers, and pharmacies, among many others. Until recently, there was limited outreach to these partners, and for the most part they have not been very engaged in preparedness activities. Hurricane Sandy showed how essential these parts of the health sector are to resilience. When these services failed during the hurricane, patients turned to the overburdened hospitals.

➢ **Novel funding mechanisms should be sought to incentivize community-based, grassroots organizations to become more engaged in preparedness activities in their communities.**

Businesses, philanthropies, and other private entities should be engaged as partners with governments in promoting disaster resilience at the community level. The president should declare that community resilience is a national priority and launch an initiative to kick-start such efforts. The federal government should pilot incentive programs to communities and community-based organizations to foster disaster resilience work at a grassroots level. One possible path is to experiment with competitive grants to local organizations funded through a combination of federal, state, private, and philanthropic sources.

➢ **ASPR should pilot an initiative to designate disaster specialty hospitals that can serve as centers of excellence in disaster care delivery and innovation**.

Because good preparedness is expensive, it may be more cost-efficient to consider a tiered system of preparedness in which every hospital and every community has a baseline level of preparedness but certain large institutions are designated as regional disaster specialty facilities. This is consistent with the way that trauma care is organized and with the tiered model that was developed for treating Ebola in the United States. It is important, however, that such an initiative not divert funding from the current HPP mission. A pilot project to assess the feasibility and cost of such a tiered national model should be considered.

This pilot project should be designed in collaboration with the Joint Commission and other relevant stakeholders. Competitive awards should be made to a limited number of highly motivated institutions.

➢ **States should create crisis standards of care plans informed by national guidance that include allocation of scarce resources.**

Despite the considerable progress to date, the nation remains inadequately prepared for a catastrophic event that sickens or injures tens of thousands—like a major earthquake, large-scale bioattack, or nuclear detonation. Much more work can be done to mitigate the consequences of such an event but it is likely that in any scenario like this, more people will need life-sustaining resources than there are resources available. Figuring out how best to allocate such scarce resources and implement crisis standards of care requires ongoing efforts across the country. While national guidance, such as that issued by the IOM^13^ or the American College of Chest Physicians,^14^ can be invaluable, plans must be implemented at the state and local levels. In addition to writing plans, considerable community engagement is needed to ensure that the public and healthcare providers are aware of such plans and have an opportunity to have input.

The drafting of these plans should be accompanied by community engagement efforts to educate the public and seek its input.

## Building on Success

America is stronger and safer if its healthcare and public health systems are well prepared and its communities are resilient. The substantial progress that has occurred over the past 15 years can be built upon by continued investments in programs that have proven to be successful and by experimenting with some new approaches as outlined above.
